# Modelling the effects of topographic heterogeneity on distribution of *Nitraria tangutorum *Bobr. species in deserts using LiDAR-data

**DOI:** 10.1038/s41598-023-40678-5

**Published:** 2023-08-22

**Authors:** Huoyan Zhou, Linyan Feng, Liyong Fu, Ram P. Sharma, Xiao Zhou, Xiaodi Zhao

**Affiliations:** 1 School of Ecology and Environment Science, Yunnan University, Kunming, 650031 Yunnan Province People’s Republic of China; 2 Research Institute of Forestry Policy and Information, Chinese Academy of Forestry, Beijing, 100091 People’s Republic of China; 3 Institute of Forest Resource Information Techniques, Chinese Academy of Forestry, Beijing, 100091 People’s Republic of China; 4 Institute of Forestry, Tribhuvan University, Kritipur, Kathmandu, 44600 Nepal; 5 International Center for Bamboo and Rattan, Key Laboratory of National Forestry and Grassland Administration, Beijing, 100091 China; 6 Faculty of Forestry, The University of British Columbia, Vancouver, BC V6T 1Z4 Canada

**Keywords:** Ecological modelling, Plant ecology

## Abstract

Microclimate ecology is attracting renewed attention because of its fundamental importance in understanding how organisms respond to climate change. Many hot issues can be investigated in desert ecosystems, including the relationship between species distribution and environmental gradients (e.g., elevation, slope, topographic convergence index, and solar insolation). Species Distribution Models (SDMs) can be used to understand these relationships. We used data acquired from the important desert plant *Nitraria tangutorum *Bobr. communities and desert topographic factors extracted from LiDAR (Light Detection and Ranging) data of one square kilometer in the inner Mongolia region of China to develop SDMs. We evaluated the performance of SDMs developed with a variety of both the parametric and nonparametric algorithms (Bioclimatic Modelling (BIOCLIM), Domain, Mahalanobi, Generalized Linear Model, Generalized Additive Model, Random Forest (RF), and Support Vector Machine). The area under the receiver operating characteristic curve was used to evaluate these algorithms. The SDMs developed with RF showed the best performance based on the area under curve (0.7733). We also produced the *Nitraria tangutorum *Bobr. distribution maps with the best SDM and suitable habitat area of the Domain model. Based on the suitability map, we conclude that *Nitraria tangutorum *Bobr. is more suited to southern part with 0–20 degree slopes at an elevation of approximately 1010 m. This is the first attempt of modelling the effects of topographic heterogeneity on the desert species distribution on a small scale. The presented SDMs can have important applications for predicting species distribution and will be useful for preparing conservation and management strategies for desert ecosystems on a small scale.

## Introduction

The Ulan Buh Desert is the 8th largest desert in China, and is located in the mid-latitudinal zone. It is characterized by a typical continental climate with little precipitation. The desert is located in a transitional zone with a temperate climate ranging from semiarid to arid conditions^1^. It is one of the driest regions at similar latitudes in the world and has a very sensitive ecological environment, which creates a fragile desert ecotone. The desert ecotone is the key ecoregion and is suitable for researching ecosystem degradation and recovery mechanisms. Desert ecotones may have high ecological significance, which quantitatively reveals the interactions between plants and environmental factors. The spatial distribution pattern of the desert plants is therefore the result of the interactions between the plant communities and the harsh environmental factors^2^.

Spatial distributions are fundamental to species ecology^3^ and help identify threats to the conservation of plant species, manage the impacts of biological invasion^4^, and plan conservation strategies. Climate, edaphic, and topographic factors are the main factors affecting species distributions^5^. These factors are affected by a variety of biophysical processes including competitive interactions between plant species and their dispersal history^6,7^. SDMs, like habitat suitability or ecological models, are developed with the observed species occurrence as a response variable and environmental factors as predictor variables^8^. Many modelling techniques are available to developed SDMs^9–11^. Although previous SDMs studies have compared different modelling techniques for their precision^12–17^, most of them describe species distributions over a large scale, and therefore have low predictive performance in small-localized condition^6,18–20^. Most SDMs are calibrated only with climate data at a low spatial resolution, such as the climate data acquired from WorldClim(https://www.worldclim), with a 1 km^2^ grid resolution. However, some studies have indicated that the predictive performance of SDMs can be substantially improved by incorporating other environmental factors in addition to climate factors^21^ or by considering finer-scale processes, such as topo-climatic processes^22^. Topographic heterogeneity is the most important factor affecting the spatial distribution of desert plants^23^. In the mountainous regions, topography may precisely describe local temperature, light, and humidity, Topography significantly affects light and water availability and soil development. Furthermore, topography affects species composition, structure, appearance, and dynamics of the plant communities^24–26^.

Building on the previous work done in this field, the current study intends to answer a few important questions including ‘What distribution patterns are followed by the desert plant species?’ and ‘Which topography factors play a major role in affecting the species distribution?’ Intense solar radiation, high evapotranspiration, extreme temperatures, degraded soil and vegetation cover, low atmospheric moisture, and harsh topographic features are important factors in the desert environment. The desert is extremely arid with < 60–100 mm mean annual precipitation, and therefore, its moisture regime is the most critical factor for regulating biological processes and species distribution. The rainfall spatially varies at different scales, not only at the regional scale ranging from 0.1 to 10 km, but also at a local scale of few meters. Rainfall is the main source of moisture for desert plants, which triggers species' survival and growth^27^. Many studies have explored the appropriate methods for understanding the relationships between vegetation dynamics, soil moisture, and temperature in the desert ecosystem^28–30^. However, under similar climate and desert environmental conditions, the influence of topography on species distribution has rarely been investigated in a small scale.

Additionally, several studies have been conducted on the topographic factors affecting animal communities in the desert. In recent decades, some studies have been conducted to model the environmental variables influencing kit fox distribution. For example, Maxent fixed-effects and generalized linear mixed-effects models have been developed to describe kit fox distribution^30^. These modelling studies show the large effects of elevation on kit fox survival and growth, followed by that of the slope and canopy height of the vegetation at the landscape scale. The Maxent algorithm was used to predict the habitat in the Mojave desert and parts of the Sonoran desert considering the geographical and topographical factors at the spatial grid of one square kilometer^31^. The field-validated site—and landscape-level SDMs were developed to identify the potentially rare and endemic plant habitats in the Great Basin of Western North America^32^, in which the potential habitat combined with the elevation, slope, aspect, rock type, and geologic processes are mapped with the resolution of 100 square meters. Similar to climatic factors, non-climate predictors (e.g., topography and habitat) are also important at a finer scale^33^. Thus, the analysis of these factors on a finer scale could be the most appropriate for developing SDMs and increasing their predictive accuracy^34^. As mentioned above, topography is one of the most important predictors of SDMs. It controls the habitat structure, biota, and all growing conditions locally^35^, especially in the desert, where topographic features are more unfavorable than other land features. Thus, a high-resolution topographic map is necessary to develop SDMs.

For dominant species, such as *Nitraria tangutorum *Bobr. in the desert, a small geographic range and narrow habitat may lead to acute habitat limitation, which is strongly correlated with other environmental factors. The microclimate, which influences ecosystem dynamics and processes, is often disregarded in ecology and evolution^36^. It is meaningful to investigate the geographical variations of *Nitraria tangutorum *Bobr. species in the desert on a small scale, such as within the scale of 100 m, where topographic factors significantly affect species distribution. Existing studies have shown that geographic and topographic predictors can substantially improve the fitting performance of SDMs^37^.

High-resolution terrain data are rarely available. Remote sensing technology, which offers alternative solutions to various data sets, can supply accurate high-resolution terrain data using the light detection and ranging (LiDAR) technology. Remote sensing data is important for determining species distribution^38,39^. In recent years, LiDAR datasets have become useful for modelling biodiversity and habitat analyses. For example, LiDAR data could improve the prediction of composition and changes in the plant communities^40,41^, improve the descriptions of animal behaviors and their spatial distributions^42^, and enhance the species richness prediction^17^.

In this study, we developed SDMs for revealing the relationships between desert plants and topographic factors affecting species distributions. Topographic variables used in the SDMs were derived from LiDAR data (resolution: 0.15 m). The SDMs could be used to predict the precise distribution of species covering an area of one square kilometer with a resolution of 10 m. Different modelling algorithms proposed in this study were evaluated based on the dataset obtained from 67 *Nitraria tanguotrum* species in the Ulan Buh Desert, an arid land in northern China. The objectives of this study were to: (1) compare various modelling algorithms including parametric and non-parametric modeling based on statistical indexes, and determine the best to develop SDMs; (2) evaluate the contribution of topographic factors to the precise prediction of distribution of the plant species of interest in the desert; (3) determine the effects of topographic heterogeneity on the distribution of *Nitraria tangutorum Bobr;* and (4) present the habitat suitability map for *Nitraria tangutorum Bobr*. and show the corresponding management implications of the presented SDMs.

## Results

### Model performances

**Figure 1:**
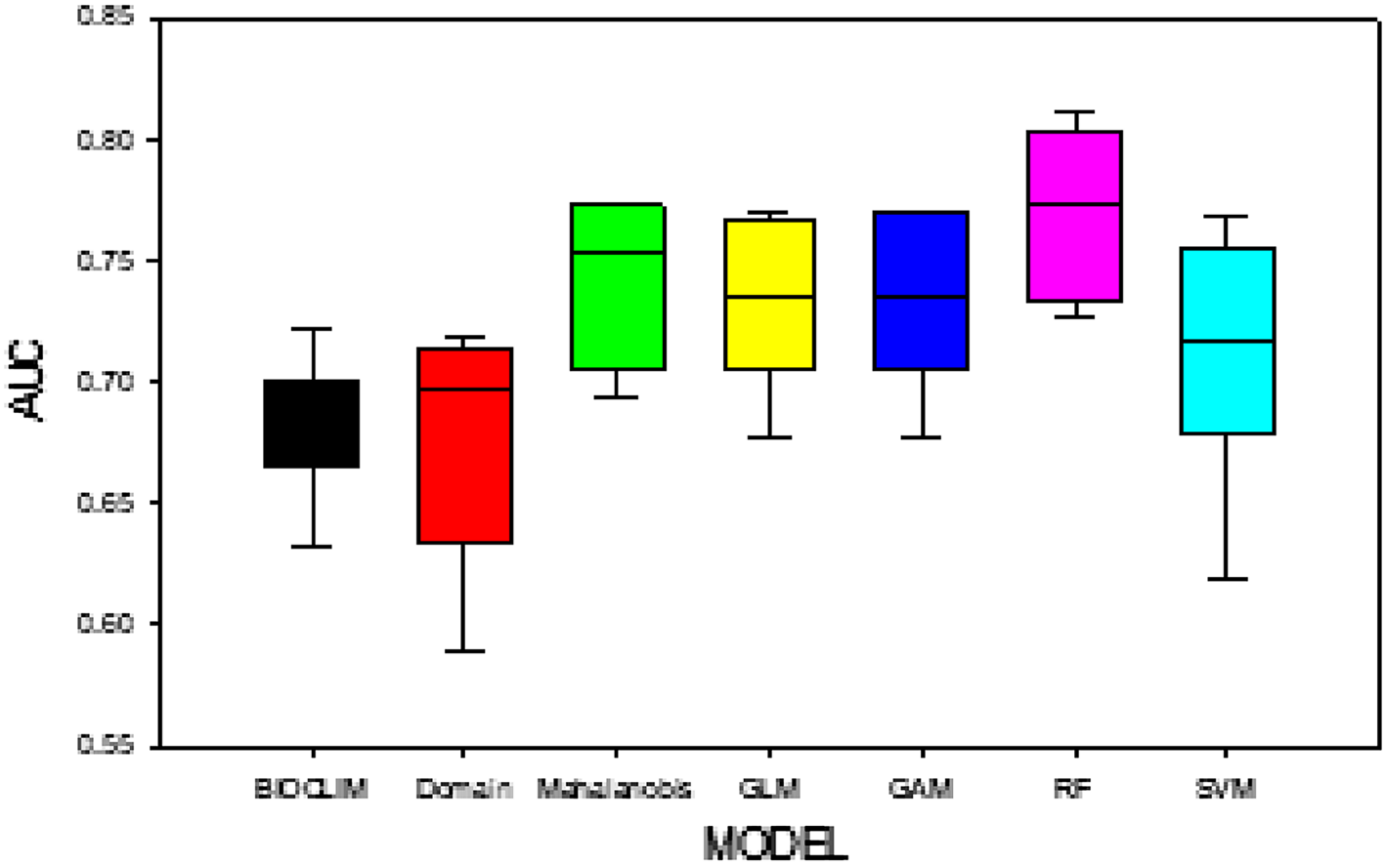
The boxplots of different models (BIOCLIM: classic climate envelope model; Domain computes the Gower distance between environmental variables and locations of occurrence; Mahalanobis model based on the mahalanobis distance; GLM: Generalized Linear Model; GAM: Generalized Additive Model; RF: Random Forest; SVM: Support Vector Machine).

Random sampling and each of the seven models were run ten times, and the Area Under Curve (AUC), true positive rate (TPR), and false positive rate (TNR) were calculated. With the presence of 67 and the absence of 100, the results indicated the AUC varied from low (0.59) to high (0.81), indicating different qualities of the prediction SDMs of *Nitraria tangutorum *Bobr. (Fig. 1). The RF produced the highest AUC value, and the Domain had the lowest AUC value (Table 1). All the model performances were fair and satisfactory, except for BIOCLIM and Domain. RF produced the largest AUC, but not with maximum TPR and TNR; TPR and TNR of RF were the third lowest of seven models. Mahalanobis produced the highest TPR + TNR value.

**Table 1:** Evaluation measures of seven species distribution models.

Run time	Model evaluation	BIOCLIM	Domain	Mahalanobis	GLM	GAM	RF	SVM
	Number of presences	67	67	67	67	67	67	67
	Number of absences	100	100	100	100	100	100	100
1	AUC	0.6767	0.7178	0.7741	0.7704	0.7704	0.8043	0.7681
	TPR + TNR	0.1402	0.6595	0.6799	0.4836	0.4836	0.4565	0.7167
2	AUC	0.6689	0.7093	0.7555	0.7631	0.7704	0.8111	0.7477
	TPR + TNR	0.1521	0.6370	0.6560	0.4211	0.4836	0.4221	0.1208
3	AUC	0.6808	0.6870	0.6949	0.7345	0.7345	0.7321	0.7169
	TPR + TNR	0.0626	0.6209	0.5793	0.2996	0.2996	0.2884	0.1788
4	AUC	0.7227	0.6978	0.7531	0.7396	0.7396	0.7880	0.7337
	TPR + TNR	0.1700	0.7222	0.6364	0.4825	0.4825	0.4647	0.3292
5	AUC	0.6325	0.6067	0.6932	0.7256	0.7256	0.7349	0.6713
	TPR + TNR	0.0805	0.6565	0.8508	0.4108	0.4108	0.2795	0.1636
6	AUC	0.6619	0.5888	0.7218	0.6907	0.6907	0.7275	0.6184
	TPR + TNR	0.1103	0.6253	0.6858	0.4211	0.4211	0.2051	0.0842
7	AUC	0.6962	0.6616	0.7157	0.7191	0.7191	0.7679	0.6988
	TPR + TNR	0.1402	0.6376	0.6858	0.4211	0.4211	0.3134	0.1195
8	AUC	0.7055	0.7006	0.7717	0.6766	0.6766	0.7740	0.6857
	TPR + TNR	0.2298	0.5183	0.5368	0.4220	0.4220	0.4669	0.4281
9	AUC	0.6767	0.7178	0.7741	0.7704	0.7704	0.8037	0.7638
	TPR + TNR	0.1402	0.6595	0.6799	0.4836	0.4836	0.4687	0.7061
10	AUC	0.6334	0.6264	0.7375	0.7173	0.7173	0.7897	0.7103
	TPR + TNR	0.2686	0.4677	0.3156	0.3961	0.3961	0.2720	0.2367
	TPR + TNR (average)	0.1495	0.6204	0.6306	0.4242	0.4304	0.3637	0.3084
	AUC (average)	0.6755	0.6714	0.7392	0.7307	0.7315	0.7733	0.7115

BIOCLIM: classic climate envelope model; Domain: computes the Gower distance between environmental variables and locations of occurrence; Mahalanobis: model based on the Mahalanobis distance; GLM: Generalized Linear Model; GAM: Generalized Additive Model; RF: Random Forest; SVM: Support Vector Machine; A: true positive (TP); A + B: positive (P); B: false negative (FN); C + D: negative; C: false positive (FP); D: true negative (TN); TPR: true positive rate = A/(A + B) = TP/(TP + FN) = TP/P; TNR: False positive rate = C/(C + D) = FP/(FP + TN); Rating of the model accuracy: excellent = AUC > 0.9, fair = 0.7 < AUC < 0.9, and poor = AUC < 0.7 (Swets, 1988).

For the profile models, Mahalanobis produced the highest AUC, while AUC for Domain, which was the lowest, and AUC for BIOCLIM were more stable (Table 1). For GLM and GAM, the AUC values were almost identical. For the machine learning models, AUC for RF was higher than that for SVM, and the same was true for max TPR + TNR. RF proved to be better than SVM in modelling the distribution of *Nitraria tangutorum *Bobr. in the desert.

### Habitat suitability and prediction maps for *Nitraria tangutorum *Bobr. distribution

We produced the maps (Sup. Figures 1–7) using R 3.4.3 and ARCGIS 10.5, which show the habitat suitability for *Nitraria tangutorum *Bobr. The potentially suitable habitat distribution maps of *Nitraria tangutorum *Bobr. in the study area were obtained from different algorithms. Furthermore, the corresponding species' presence/absence with each algorithm was mapped. The *Nitraria tangutorum *Bobr. species distributed mainly in the eastern part of the study area. The spatial distributions of *Nitraria tangutorum *Bobr. simulated by the different models were significantly different. Concerning the most suitable area for *Nitraria tangutorum Bobr*, the most suitable habitat area simulated by the Domain model was the largest, and the most suitable habitat area simulated by BIOCLIM model was the smallest. The suitable habitat areas simulated by GLM and GAM models were identical; the suitable habitat simulated by GLM and GAM was the largest, while that simulated by BIOCLIM was the smallest.

### The driven factors of topography on the *Nitraria tangutorum *Bobr. distribution

The responses of the driven factor- topography with different models were compared for *Nitraria tangutorum *Bobr. Different models delineated the topography characteristics of the potential distribution areas. With BIOCLIM, the existence probability followed a trend of increase at first and then decreasing trend, and aspect of the south part was on the top. For DEM, the existence probability trend of the species was steeper, reaching a peak at an altitude of approximately 1010 m. For the profile factor, the species *Nitraria tangutorum *Bobr. appeared more suitable at the predicted value of zero, and was also suitable for slope of 0°–20°. With the Domain model, the existence probability trend of the species was similar to that of the BIOCLIM model, but it seemed steeper. In the Malanhanobis model, at the aspect of the north by east 10°, the species *Nitraria tangutorum *Bobr. exhibited minimum fitness, and at the altitude range of 1007–1013 m, exhibited maximum fitness. Regarding the topography factor of the profile, there were two crests at the predicted value near zero. With the GLM and GAM models, the existence probability trend of the species was nearly the same as the linear trend line, but in the RF, the trend was nonlinear. Regarding the factor of aspect, predicted value fluctuated between 0.4 and 0.8, but for DEM factor, predicted value was the highest at an altitude of approximately 1010 m. With the factors of profile and slope, at the degree of zero, predicted value reached the top, at a slope of 20°–60°, exhibiting a rising tendency.

Analysis of the maps showed the distribution of the species *Nitraria tangutorum *Bobr. was more suitable in the south, with an altitude of approximately 1010 m, from a slope between 0° and 20°, and general trend was the same with different models. The GLM or GAM was found inadequate for the distribution modelling of *Nitraria tangutorum *Bobr.

## Discussion

### Importance of LiDAR data and its ecological implications with selected predictor variables

How does climate change affect organisms? In the past, our limited ability to map and monitor the microclimatic variations using modelling through the incorporation of the microclimate. The microclimate sensors set in the networks provided point-based approaches and weather stations provided macroclimate data. However, means of interpolating and downscaling data were lacking^43^. LiDAR is valuable for mapping microclimates, as it provides spatially continuous and sub-meter-scale information about ground topography^44^. Aerial photography provides an approach for assessing topography. However, these are less accurate than LiDAR at deriving terrain elevation^45^. An important advantage of UAVs is that they are flexible, enabling the collection of time series of aerial imagery over a period at a reasonable cost. In our study, LiDAR data were used to obtain DEM for SDMs, thereby supporting more effective conservation monitoring, management, and policy decision for a sustainable future^46^.

Our results showed that *Nitraria tangutorum *Bobr. species are specialists in the range of their habitat resources. In other words, *Nitraria tangutorum *Bobr. has a relatively narrow ecological niche.

It is limited to making the distribution by considering only topographic variables, the role of habitat heterogeneity in the desert ecosystem may be underestimated. If other environmental factors, such as light, soil, and water are considered, niche theory may be able to better explain the existence mechanism of species. Therefore, future studies need to consider more bio-variables to simulate the distribution of the species, and a permanent plot should be designed to contain the entire spatial gradient of the microclimate conditions in the study system. Long-term microclimate data series are required to complement the dynamics of the microclimate around the species. Gathering georeferenced microclimate data from different habitat types across the globe would significantly promote progress in microclimate ecology^47^. UAVs equipped with multispectral or hyperspectral sensors and laser scanner systems are very promising, providing high-resolution data in the context of microhabitats, vegetation structures, and topography^48^.

### Suggestion for improvement of SDMs

In our study, not only AUC of the models but also the responses of the variables with the models were different. The parametric regression-based models (GLM and GAM) had lower mean performance, whereas nonparametric or machine learning-based models (RF and SVM) were more accurate. GLMs are widely used in SDM studies^49^, but have poor nonlinear response performance. At the same time, the results confirmed the effectiveness and robustness of the machine-learning techniques. Similarly, Marmion et al. found a single algorithm with lower accuracy than RF in predicting the plant distributions^50^. The parameters of the model affect its accuracy; for example, the performance of RF depends on two important parameters, ntree and mty. As for the ntree, the optimal value using only the default may not be the best; therefore, future research should test and adjust the model parameters to the optimum state. In this study, various parametric and nonparametric algorithms were evaluated for SDMs. In the next step, semi-parametric models^51^ shall be used for better results.

A stable and reliable mathematical algorithm is required to develop more accurate SDMs. In previous studies of community-level predictions of the species distributions, the choice of how species and site-level occurrence probabilities are combined into species assemblage predictions is more important than the choice of the model type used^52^. Following research shall use ensemble models to predict species distribution. As many researchers have expressed, no evidence has proven that one model is steadily accurate, but it is proven to be better than other models.

The number of environmental factors that accurately represent the habitat characteristics of a species was sufficient SDM has some limitations, for example, over-prediction of species richness per site and sensitivity to methodological biases. In addition, sample size, sample prevalence, sample design, model techniques, imperfect detection of species, or the choice of environmental variables could affect the uncertainty of the predictions^53^.

The relationships between species occurrence and sets of predictor variables explored by the models produce two kinds of useful outputs: estimates of the probability that species might occur at a given unrecorded location^50^, and estimates of an area’s suitability for species ^16^. Climate determines the distribution of desert species^54^. Previous research has shown that the roots of desert plant species develop and depend on the underwater^55^. As the study area was only one square kilometer, the variable of climate change could not be obvious, and species distribution could have been disturbed by the level of underground water. However, further studies are necessary in this regard. It is well known that biotic interactions play an important role in creating accurate SDMs for many species, particularly at small geographic scales, such as host requirements and competition^6,56^. In this study, the model did not include the interaction between species and the influence of predators or human activities. More efforts should be dedicated to include fine-scale environmental measures, such as micro-temperature, underwater, moisture, and rainfall, for fine-scale species mapping and management.

### Management implication of SDMs

Species conservation and management are key to maintaining regional ecological balance, especially in ecologically fragile areas such as the Ulan Buh Desert. This study could distinguish the habitat requirements of *Nitraria tangutorum *Bobr., especially for the topography, to provide a reference for protection. *Nitraria tangutorum *Bobr. was found to be distributed mainly in the eastern part of the study area. This finding is consistent with the results of the actual survey. The suitable habitat area is larger than the real area, which indicates that the area of *Nitraria tangutorum *Bobr. is more widely distributed under current environmental conditions. Some of the suitable habitat characteristics shown in our study were consistent with those of other studies^57^. For example, the population size of *Nitraria tangutorum *Bobr. increases with sand depth, making it suitable for dunes. To expand the area of *Nitraria tangutorum *Bobr., the land should be dune, and will be a good choice at an altitude of approximately 1010 m, better to face the aspect of the south part, within the slope of 20°. Because of this characteristic, *Nitraria tangutorum *Bobr. can prevent quicksand from moving forward and land desertification. *Nitraria tangutorum *Bobr. is an excellent species for sand fixation.

## Materials and methods

### Study workflow

**Figure 2:**
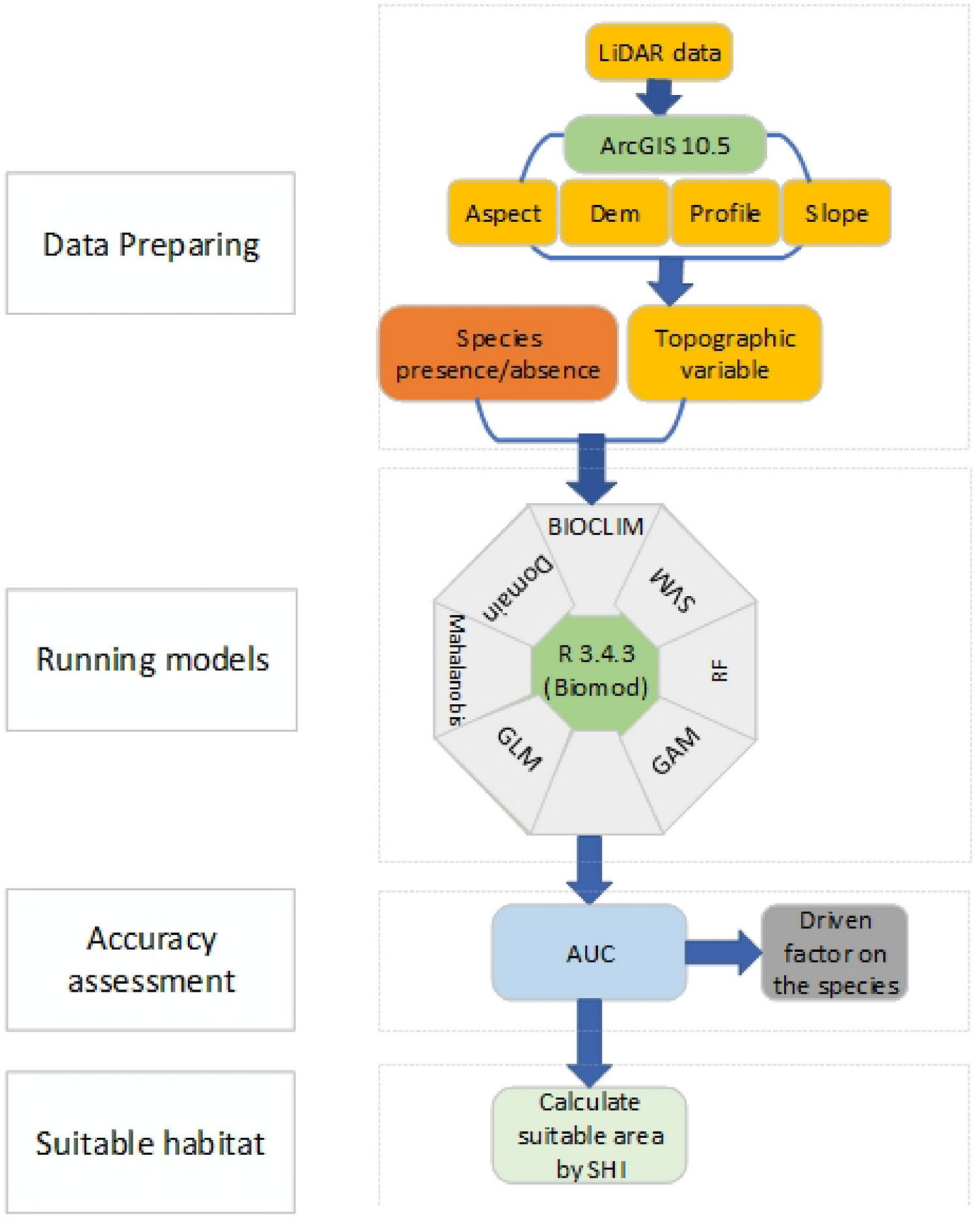
Workflow of the study. ARCGIS 10.5 is the software of geographic information system, R3.4.3 is the data processing platform, AUC is the area under the receiver operator curve, which evaluates the fitted models. Species distribution models include Bioclimatic Modelling (BIOCLIM), Domain, Mahalanobis, Generalized Linear Model (GLM), Generalized Additive Model (GAM), Random Forest (RF), Support Vector Machine (SVM). SHI denotes suitable area index.

The study process shown in the flowchart (Fig. 2) consists of four parts: (1) preparing data, including species data and factors of topography; (2) fitting different models; (3) evaluating models by Area Under the Curve (AUC) and comparing the driving factors of different models; and (4) estimating areas suitable for habitat using the developed SDMs.

### Study area and data analysis

#### Study area

**Figure 3:**
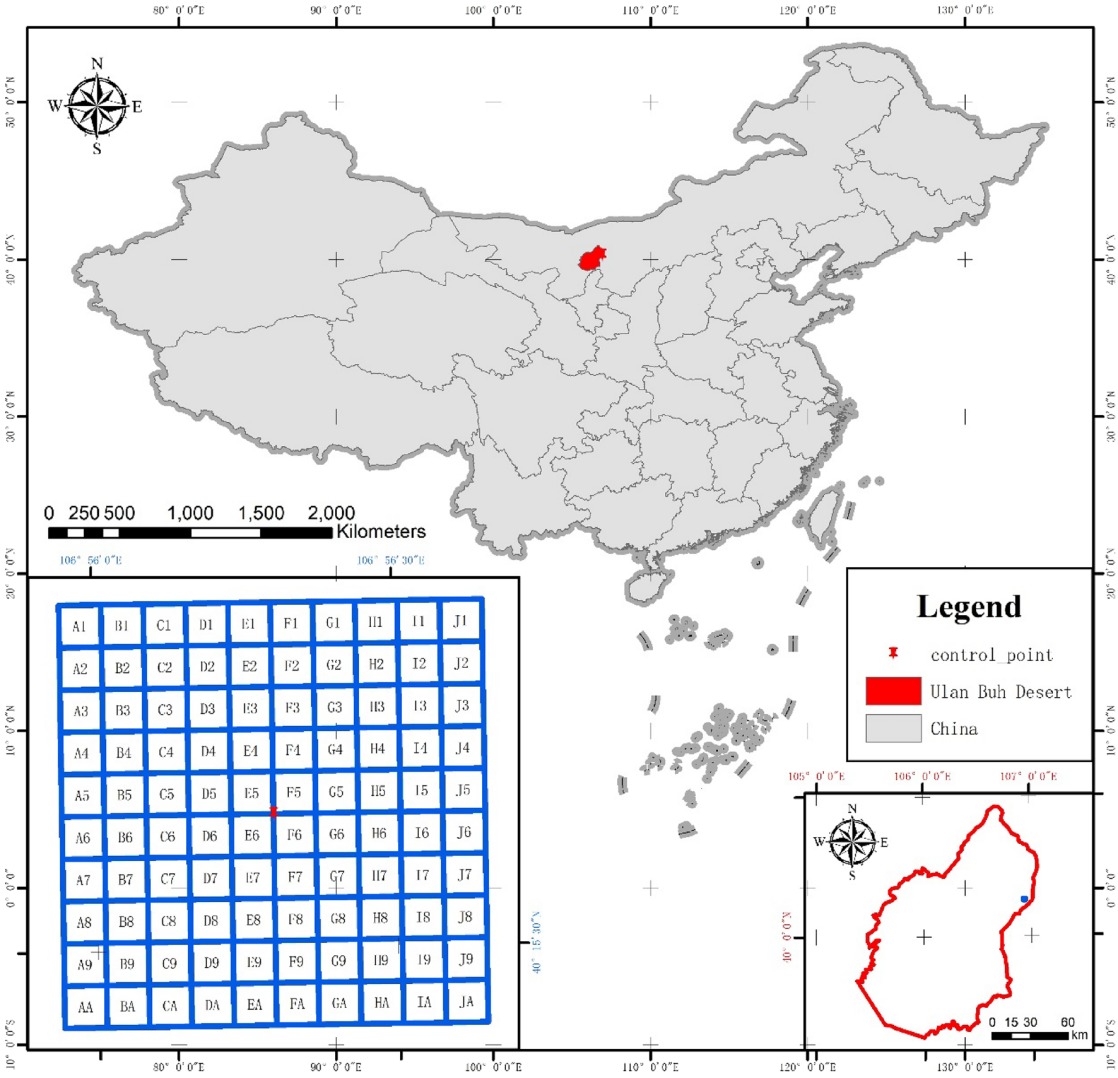
Location of study site in the Ulan Buh desert (the top of the figure is the northwest arid area in China; the bottom left is the study area located in Ulan Buh desert; and the bottom right is the plot of Ulan Buh desert. The number of rows are 0,1,2,3,…and the columns are A,B,C,…in the proper sequence).

The Ulan Buh Desert is located in the northern part of Inner Mongolia Province, 106°38′42″E—106°57′00″ E, 40°17′24″ N–40°28′36″ N (Fig. 3), covering an area of 14,905.13 km^2^. The elevation ranges from 1009 to 1016 m, and the topographical profile on the surface varies from 1211.58 to 1339.63 m^58^. The climate is characterized by semi-arid to arid conditions, with a mean annual precipitation of 147.4 mm, mean annual evaporation of 2458.4 mm, mean annual relative humidity of 47.3%, and mean annual temperature of 6.8 °C in the desert^59^. The eastern edge of the desert is important for dividing the desert and grassland in Central Asia. Our study was based on control plots, such as desertification processes and control treatments^60^.

#### Species data processing

*Nitraria tangutorum *Bobr. is a shrub species. It is 1–2 m tall, multi-branched, crooked, prone, and spreads with infertile needle-like apex branches and white tender branches. The leaves are clustered in 2–4 pieces on the young shoots, broadly lanceolate, 18–30 mm long, 6–8 mm wide, blunt apex, base narrowed into a wedge, and entire rare apex lobed. The flowers are densely arranged. The drupe is oval and dark red when ripe, juice rose-colored, 8–12 mm long, and 6–9 mm in diameter. (http://www.iplant.cn/) (State Key Laboratory of Systems and Evolutionary Botany, Institute of Botany, Chinese Academy of Sciences). Supplementary material Fig. 8 for photo of plant *Nitraria tangutorum Bobr* in the field investigation.

(1) Species data collection

The ground surveys were conducted between August and September 2019. 100 sample plots of *Nitraria tangutorum *Bobr. plant community was established in one square kilometer at the eastern edge of the study area. Each 100 × 100 m sample plot was divided into 100 small quadrats with an area of 10 × 10 m (Fig. 3). The center points GPS location is southeast corner of the sample F5, and the accuracy of the point is within a half meter. The grids that were created in the same direction as we sampled in field, all are facing north. 335 occurrence records for *Nitraria tangutorum *Bobr. were collected in the area (Fig. 5). The formal identification of the plant material used in my study has complied with the Chinese Virtual Herbarium (CVH): (https://www.cvh.ac.cn/spms/detail.php?id=f76aceff), and a specimen of this material has been deposited in a publicly available herbarium. And we were employed by the Chinese Academy of Forestry, which is also the State Forestry Administration Dengkou Desert ecosystem positioning observation station. To ensure that we have permission to collect *Nitraria tangutorum *Bobr. We confirm that all methods were performed following current guidelines and regulations in China, Huoyan zhou, Xiao Zhou, and Xiaodi Zhao undertook the formal plant identification, and field investigation were carried out.

(2) Processing species data

The data were cleaned for use in our modelling. The longitudes and latitudes of the sample plot center were recorded as coordinates, and if the record was out of the boundary of the study area, it was checked with the UAV-RGB (Unmanned Aerial Vehicle—Red Green Blue) data and overlaid on the map. If the canopy was outside the plot boundary, the root inside was preserved.

The coordinates were cross-checked by a visual or ‘overlay’ function using ArcGIS 10.5. This study applied the coordinate function from the ‘SP’ (spatial point) package to create a Spatial Point Data Frame, and then the over function from ‘SP’ to do a point-in-polygon query with the study boundary. Moreover, the occurrence of each grid would be only once, without duplicate records; the function ’duplicate’ could be used to remove duplicates in the software R3.4.3.

The species data included the presence and absence data, the resolution of the grid was 10 m, and each grid retained only presence or absence data, reducing the bias of the model predictions by space sampling^61^.

### Environment data processing

LiDAR data

**Figure 4:**
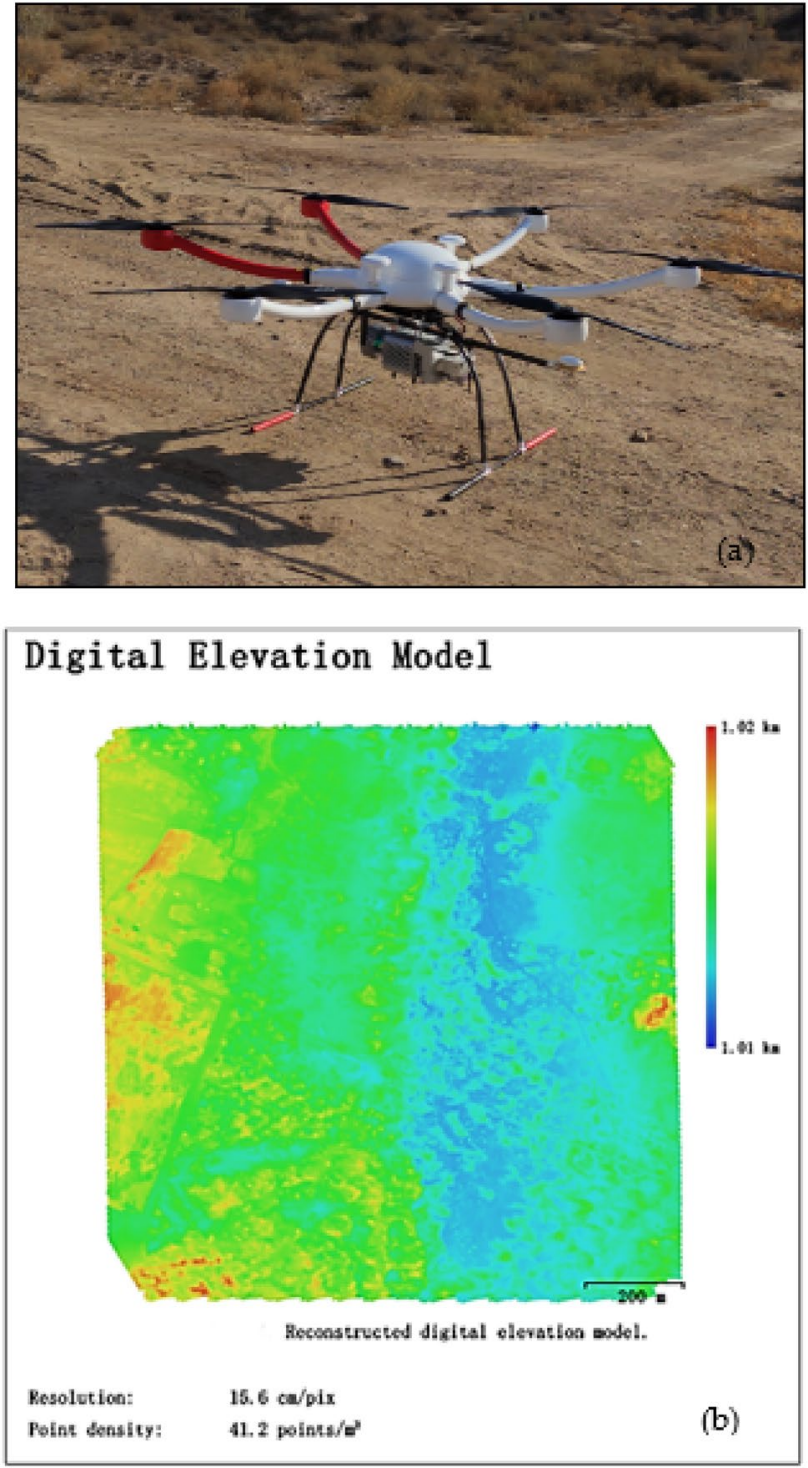
The Unmanned Aerial Vehicle (UAV) Light Detection and Ranging (LiDAR); (**a**) is the UAV LiDAR; (**b**) is the digital elevation model (DEM), and legend describes the elevation of the point.

The UAV technique with the SPAN_IGM_STIM300 IMU (Fig. 4a) was used to gather airborne LiDAR data during the study conducted on October 24, 2019. The LiDAR parameters were a pulse rate of 125 kHz, a scan angle (FOV(field of view)) of ± 22.5°, and a laser beam divergence (IFOV) of 0.5 mrad. An average point density of 41.62 points per m2 and a footprint diameter of 25 cm was obtained.

Topographic variables

**Figure 5:**
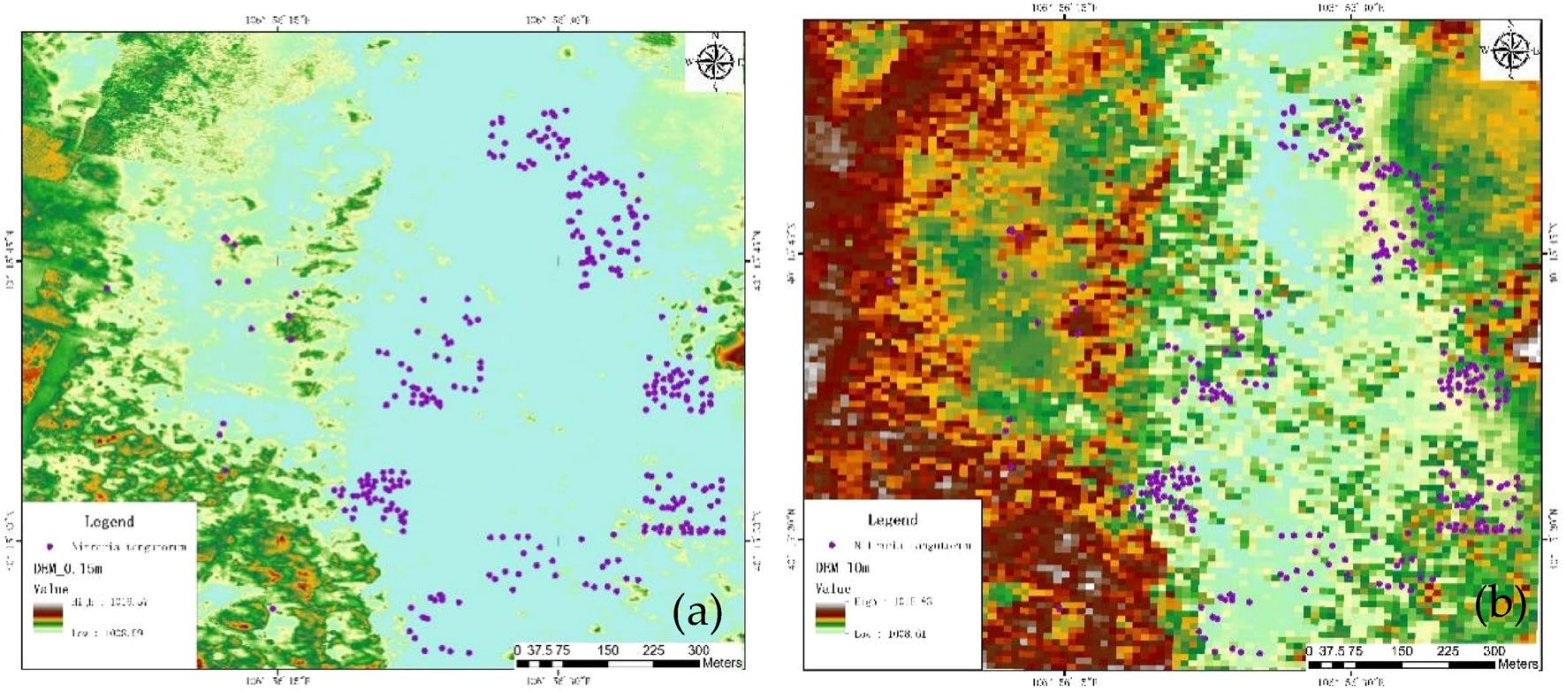
The original and rescaled LiDAR data with different resolution, (**a**) is LiDAR DEM with a resolution of 0.15 m, (**b**) is LiDAR DEM with a resolution of 10 m, and the purple points indicate the occurrence of *Nitraria tangutorum* Bobr.

**Figure 6:**
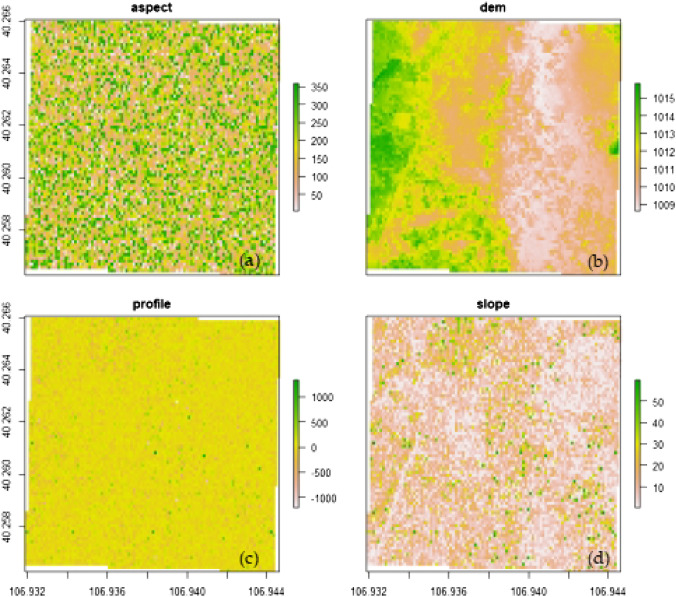
Maps of the predictor variables. The horizontal and vertical coordinates represent latitude and longitude, respectively. The (**a**) indicates different aspect of the study area, (**b**) indicates different elevation of the study area, (**c**) indicates different profile of the study area, and (**d**) indicates different slope of the study area

The digital elevation model (DEM) was derived from the UAV LiDAR point cloud (Fig. 4b) with a resolution of 15 cm (Fig. 5a) and then resampled to 10 m (Fig. 5b) with image analysis tools. Other topographic variables, including slope, aspect, and profile (Fig. 6), were obtained using the spatial analysis model in ArcGIS 10.5. Predictor variables were organized as raster (grid) files for species distribution modelling. After generating the set of predictor variables (rasters) and the occurrence points, our next step was to extract the values of the predictors at the locations of the points using the raster tools of ‘extract’ in ArcGIS 10.5. To select the significant predictor variables, the variance inflation factor (VIF) was calculated. A VIF of 10 was acceptable as it showed no significant collinearity among the predictor variables^62^. All variables were resampled using ArcGIS 10.5 to unify the space dimension of 10 m, which was the same value as the resolution of the species samples.

### Forms for SDMs

To predict species distribution, a number of statistical and machine-learning approaches have been used^63,64^. In conjunction with geographic information systems and remote sensing, the methods used in species distribution modelling can be classified as profile, regression, and machine learning methods. The profile method only considers the presence (occurrence) data, whereas the regression and machine learning methods use both the presence and absence data. The profile method includes BIOCLIM^65^, Domain^66^, and Mahalanobis^67^. In addition to these methods, we also analyzed and compared the predictive performance of SDMs developed using regression methods, such as the GLM^68^ and GAM^69,70^, and some machine learning methods, such as RF^71^ and SVM.

Sampling bias may have been present in the occurrence data used^72^. Random sampling was performed to record the presence or absence data with the random points function in the ‘dismo’ package. An ‘extent’ tool was used to further restrict the area from where random locations were drawn^8,73^. The ‘dismo’ package containing the k-fold function facilitates the data partitioning and creates a vector that assigns each row in the data matrix to a group (between 1 and k with k = 5).

We used seven modelling algorithms, including both nonparametric and parametric algorithms, to develop the SDMs, seven modelling algorithms are briefly described below.

### Bioclimatic modelling (BIOCLIM)

BIOCLIM is a classic climate envelope model^65^. Although it generally does not perform as well as some other modelling methods^74^, particularly in the context of climate change^72^, it is still being used, because the algorithm is easy to understand.

### Domain

The Domain algorithm^66^ has been extensively used for species distribution modelling. The Domain algorithm computes Gower’s metric^75^ between the environmental variables at any location and those at any of the known locations of occurrence, which were defined as training sites in this study.

A suitable means of quantifying the similarity between the two sites is provided by Gower’s metric. In Euclidean Q-dimensional space, the distance d between two points A and B is defined as

1 $${d}_{AB}=\frac{1}{Q}{\sum }_{K=1}^{Q}\left(\frac{{A}_{K}-{B}_{K}}{rang{e}_{K}}\right)$$

To equalize the contribution from each climatic attribute, Gower’s metric uses range standardization. In this application, the standardization method is preferred over variance standardization because it is less susceptible to bias arising from dense clusters of sample points. The complementary similarity measure (${R}_{AB}$) is defined as

2 $${R}_{AB}=\text{1} - {d}_{AB}$$

${R}_{AB}$ is constrained between 0 and 1 for points within the ranges used in Eq. 1 but may yield negative values for points outside this range. ${S}_{A}$ is defined as the maximum similarity between candidate point A and the combination of known record site Tm:

3 $$ S_{A} = \mathop {\max }\limits_{j = 1}^{m} R_{TjA} $$

${S}_{A}$ is evaluated for all grid points in a target area, where m denotes the number of known records, and a matrix of continuously varying similarity values is generated, which can be displayed as a grayscale, thematic, or contour map. As with all models discussed here, the values generated are not probability estimates but the degrees of classification confidence^66^.

### Mahalanobis

The Mahal function implements SDMs based on Mahalanobis distance^67^. The Mahalanobis distance considers the correlations of the variables in the dataset, and it is not dependent on the scale of measurements.

4 $$P{D}_{1}^{2}={\alpha }_{\mu v}(da{)}^{\mu }(da{)}^{v}$$

where ${\alpha }_{\mu v}$ is the dispersion, $(\alpha {)}^{\mu }$ is the mean value, $P$ is the independent variable, and ${D}^{2}$ is the sample value of the ${\Delta }^{2}$ statistic. Both the dispersion and mean values are subject to sampling fluctuations.

### Generalized linear models (GLM)

In GLM, ${X}_{k}(k=1,\cdot \cdot \cdot ,r)$ is the vector of $k$ predictor variables, which is related to the expected value $\mu =E(Y)$ of the response variable $Y$ (denote the response variable) through a link function $g(\cdot )$ (Eq. 5). The use of GLM in species distribution modelling is provided in^68^.

5 $$g(E(Y))=\alpha +{X}^{T}\beta $$

6 $$ g(\mu_{i} ) = \alpha + \beta_{1} x{}_{i1} + \beta_{2} x{}_{i2} + \cdot \cdot \cdot + \beta_{k} x{}_{ik} $$

where $\alpha$ is a constant called the intercept, and $\beta$ is the vector of $k$ regression coefficients (one for each predictor). (Eq. (6)).

It is assumed that the topographic factor is normally distributed and is defined as X, and the species distribution probability as Y; Therefore in the response distribution of GLM, which uses the “family = gaussian”.

### Generalized additive models (GAM)

GAM^69,70^ are extensions of GLMs. In GAMs, the linear predictor is the sum of the smoothing functions. This makes GAMs very flexible and they can fit complex functions. This also makes them very similar to machine-learning methods. The GAMs were implemented using the 'mgcv' package R 3.4.3.

7 $$g\left(\mu \right)={s}_{0}+{s}_{1}\left({X}_{1}\right)+{s}_{2}\left({X}_{2}\right)+\cdot \cdot \cdot {s}_{P}\left({X}_{P}\right) n={s}_{0}+{\sum }_{i=1}^{P}{s}_{i}({X}_{i})$$

where $\mu =E(Y\left|{X}_{1},{X}_{2},\cdot \cdot \cdot {X}_{p}\right.)$, n is for the linear prediction, and ${s}_{i}(\cdot )$ is the nonparametric smooth function. This model does not require any assumptions. It consists of a random component, an additive component, and a link function connecting the two parts. The distribution of the response variables(Y) belonging to the family of exponential distributions can be binomial.

### Random forest (RF)

The RF method is an extension of the regression and classification trees (CART)^71^. This method can be implemented using the “random forest” function in the package with the same package name in R 3.4.3. The function RF can take a formula, or in two separate arguments, a data frame with the predictor variables and a vector with the response. If the response variable is a factor (categorical) type, RF performs classification; otherwise, it performs regression. In species distribution modelling, because RF showed remarkable modelling results, it is considered a viable option.

1. Suppose there is a dataset $D=\left\{{x}_{i1},{x}_{i2},\cdot \cdot \cdot {x}_{in},{y}_{i}\right\}(i\in \left[1,m\right])$ (${{X}_{i}}_{m}$ is the vector of $m$ predictor variables, ${y}_{i}$ are the response variables) with feature number n, sampling with replacement can generate a sampling space $(m*n{)}^{m*n}$.
2. Build a basic learner (a decision tree): ${d}_{i}=\left\{{x}_{i1},{x}_{i2},\cdot \cdot \cdot {x}_{in},{y}_{i}\right\}(i\in \left[1,m\right])$ generates a decision tree for each sample (where K <  < M) and records the results of each decision tree ${h}_{j}(x)$.
3. Training times to T $H(x)=\mathit{max}{\sum }_{t=1}^{T}\phi (h(x)=y)$, where $\phi (x)$ is an algorithm (absolute majority voting, majority voting, weighted voting method, etc.).

RF requires two parameters: (1) mtry, the number of predictor variables performing data partitioning at each node, and (2) ntree, the total number of trees to be grown in the model run. In this study, ntree (number of trees to grow) was set to 500 and mtry to 10 after some initial tuning experiments.

### Support vector machines (SVM)

SVM^76^ applies a simple linear method to data; however, in a high-dimensional feature space, nonlinearly exists in the input space. However, in practice, this does not involve any computations in a high-dimensional space. This simplicity, combined with state-of-the-art performance on many learning problems (classification, regression, and novelty detection), has contributed to the popularity of SVM^77^. They were first used in species distribution modelling by Guo et al.^78^. There are several implementations of SVM in R 3.4.3. The most useful implementations in our context are the function 'ksvm' in the package 'kernlab' and the 'svm' function in the package 'e1071'. The 'ksvm' includes many different SVM formulations and kernels, provides useful options, and features a method for plotting, but it lacks a proper model selection tool. The 'svm' function in package 'e1071' includes a model selection tool: the 'tune' function^77^.

8 $$\widehat{y}={\{}_{-1,{\sum }_{i=1}^{m}{\lambda }_{i}^{*}{y}_{i}K\left(x,{x}_{i}\right)\le -1}^{+1,{\sum }_{i=1}^{m}{\lambda }_{i}^{*}{y}_{i}K\left(x,{x}_{i}\right)\ge +1}$$

Given the training examples, ${x}_{i}(i=\mathrm{1,2},\cdot \cdot \cdot m)$ indicates the samples to be classified, $\widehat{y}\in \left\{+1,-1\right\}$ indicates the marked label value for the samples, + 1 denotes the point above the separating hyperplane, and -1 denotes the point under the separating hyperplane, where $K\left(x,{x}_{i}\right)$ is the Gaussian kernel function, $K\left(x,{x}_{i}\right)$ is nonlinearity, and ${\lambda }_{i}^{*}$ is the optimal solution to the primal problem obtained by solving the dual problem. Note that if a sample ${x}_{i}$ is not a support vector, to maximize the Lagrangian function, there must be a λ* = 0 non-support vector corresponding to ${\lambda }^{*}$ equal to zero, which theoretically shows that the decision boundary of the SVM is only related to the support vector.

### Evaluation and comparison of SDMs

#### Accuracy assessment

Different measures may be used to evaluate the prediction ability of each model^79–81^. Many measures for evaluating models based on presence-absence or presence-only data are threshold dependent. This implies that a threshold must first be set. However, many statistics are threshold independent, such as the correlation coefficients and the area under the receiver operator curve (AUROC, generally abbreviated as AUC). The AUC value is the area of ROC and the X-axis under the ROC curve; the larger the area, the higher the relationship, and the greater the creditability. When the AUC value is greater than 0.7, the result is fair, and when the AUC is higher than 0.9, the result is excellent^82^. We applied this measure to evaluate the SDMs.

In this framework, an algorithm is supposed to predict “positive” or “negative”. However, some concepts are confusing, as summarized in Table 2.

**Table 2:** Receiver operation characteristics.

Ground truth/prediction	Positive	Negative	Rate
Positive	A	B	TPR
Negative	C	D	TNR

A: true positive (TP) ; A + B: positive (P); B: false negative (FN); C + D: negative; C: false positive (FP); D: true negative (TN); TPR: True positive rate = A/(A + B) = TP/(TP + FN) = TP/P; TNR: False positive rate = C/(C + D) = FP/(FP + TN); and Accuracy (ACC) = (A + D)/(A + B + C + D).

#### Suitable habitat index (SHI)

The probability of species occurrence was extracted using ArcGIS. The lowest probability value was selected to distinguish the suitable zone from the non-suitable zone^83^. The habitats of the entire study area were divided into three categories using the frequency statistical method^84^: unsuitable habitat (SHI ≤ 0.3), low suitable habitat (0.3 < SHI ≤ 0.5), and suitable habitat (SHI > 0.5).

## Conclusion

This is the first attempt at modelling the relationships between the spatial distribution of desert plant species and topographical factors extracted from the LiDAR data. The results indicated a general relationship between model performance and modelled species distribution on a small scale. Topographical factors should be contingent on the modelled species distribution. The LiDAR technique has certain applications in species distribution simulations. The RF produced the highest AUC when AUC was integrated into TNR + TPR (false positive rate plus true positive rate); Manhalanobis showed the best SDMs to predict the distribution of *Nitraria tangutorum *Bobr. in the desert on a small scale. The suitable habitat distribution area of *Nitraria tangutorum *Bobr. predicted in the study was larger than the actual area. A flat land with an aspect of the south at an altitude near 1010 m was found to be the driving topography of the *Nitraria tangutorum *Bobr. distribution. Thus, this study provides a reliable reference for the restoration and management of desert vegetation.

### Supplementary Information

Supplementary Figures.

## Data Availability

Data used in this study are available from the National Forestry and Grassland Science Data Center (http://www.forestdata.cn/). Data is available when needed, and Huoyan Zhou should be contacted.
