# Supplementary material for: Modelling the effects of topographic heterogeneity on distribution of Nitraria tangutorum Bobr. species in deserts using LiDAR-data
Source: Sci Rep. 2023 Aug 22;13:13673. doi: 10.1038/s41598-023-40678-5 (PMC10444836; doi:10.1038/s41598-023-40678-5)
Supplement: Supplementary file 1 — Supplementary Figures. [file 41598_2023_40678_MOESM1_ESM.docx]

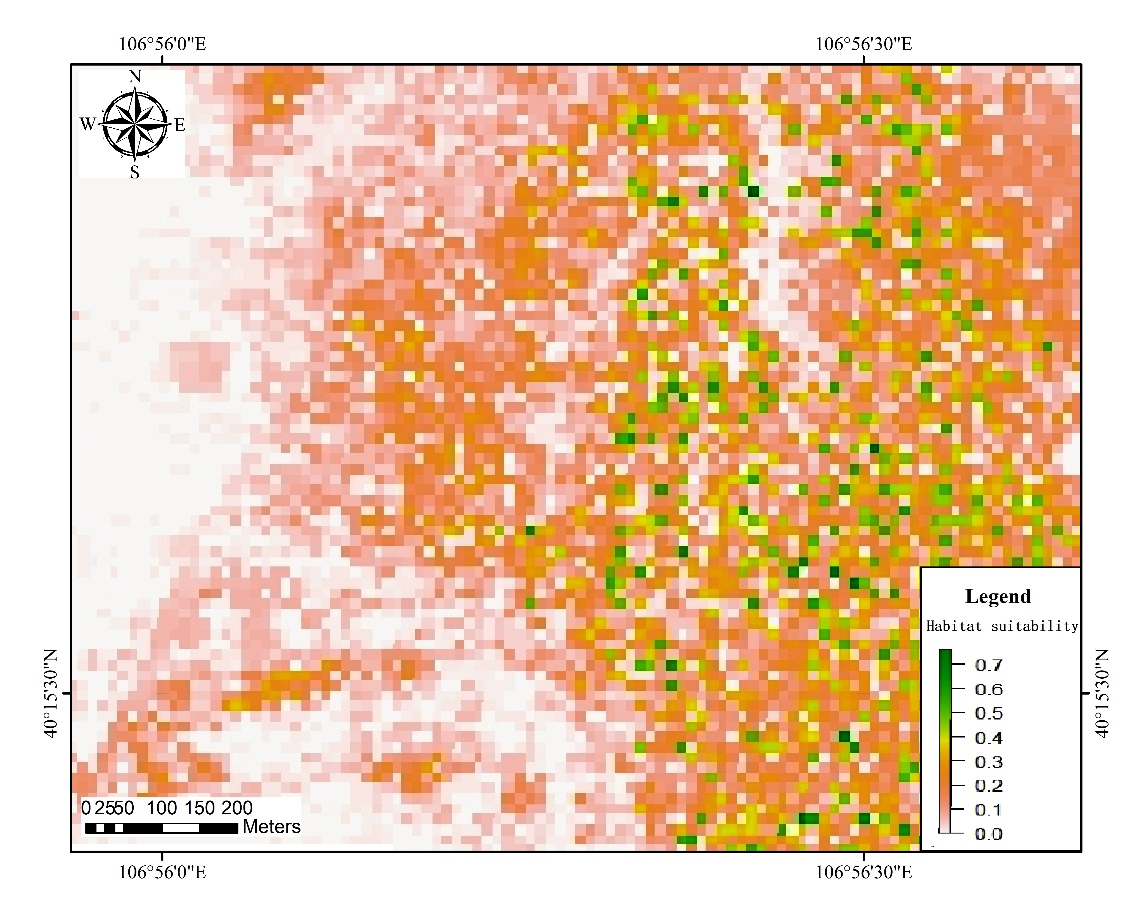


**Sup. Figure 1** Habitat suitability map produced with BIOCLIM
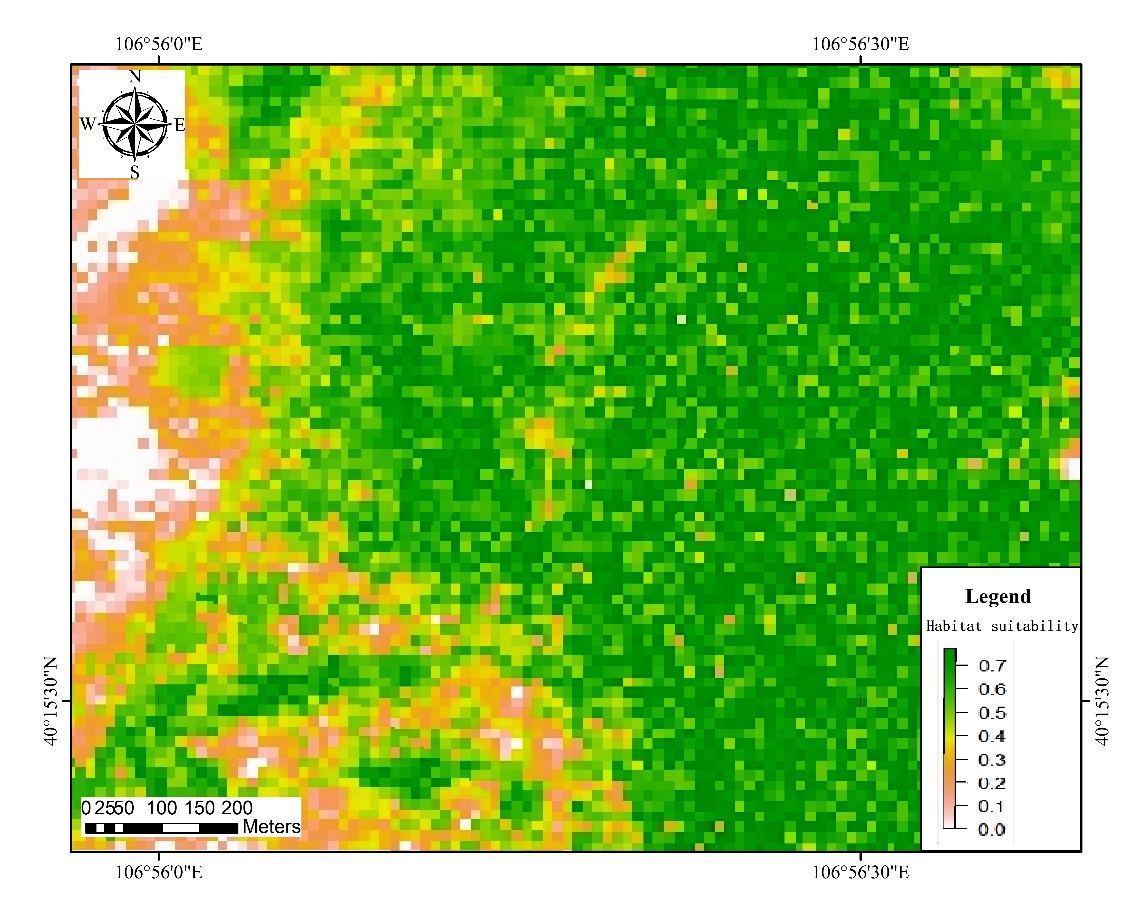


**Sup. Figure 2** Habitat suitability map produced with Domain model


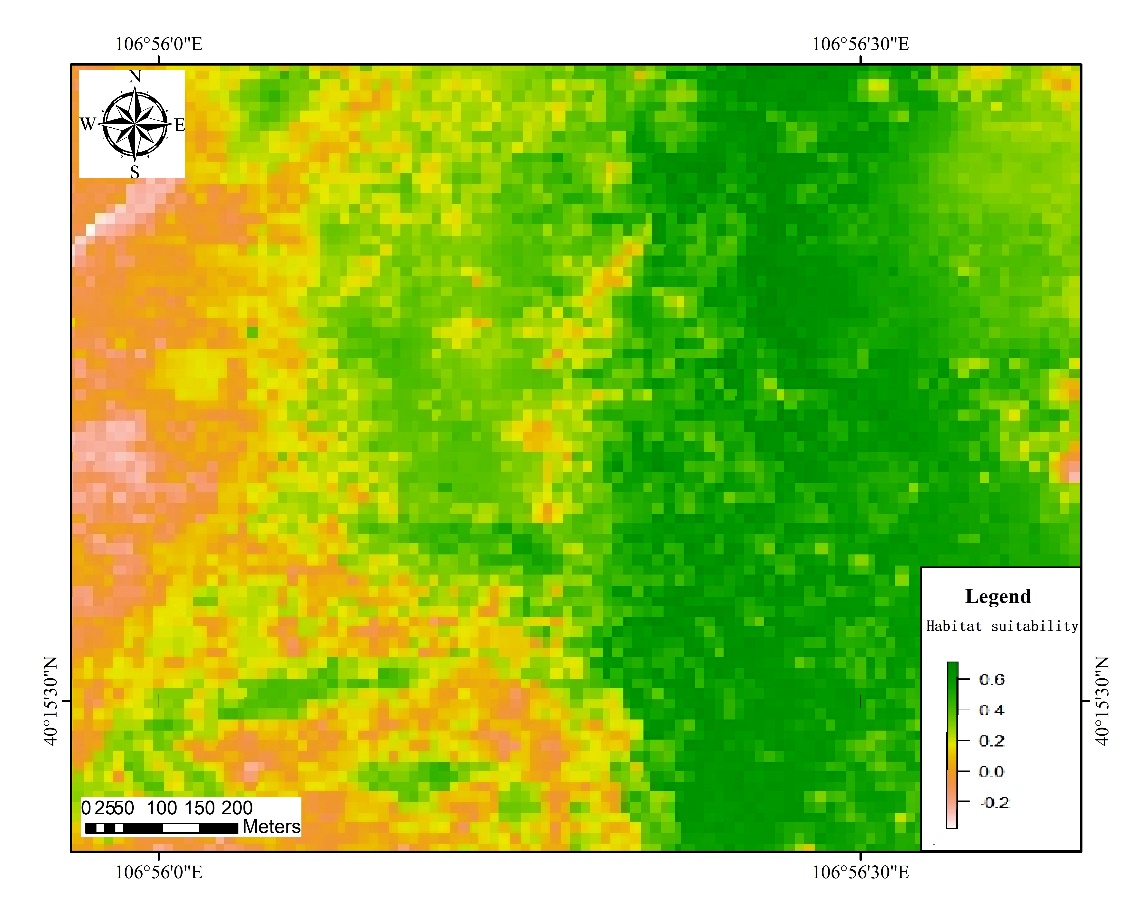


**Sup. Figure 3** Habitat suitability map produced with GAM


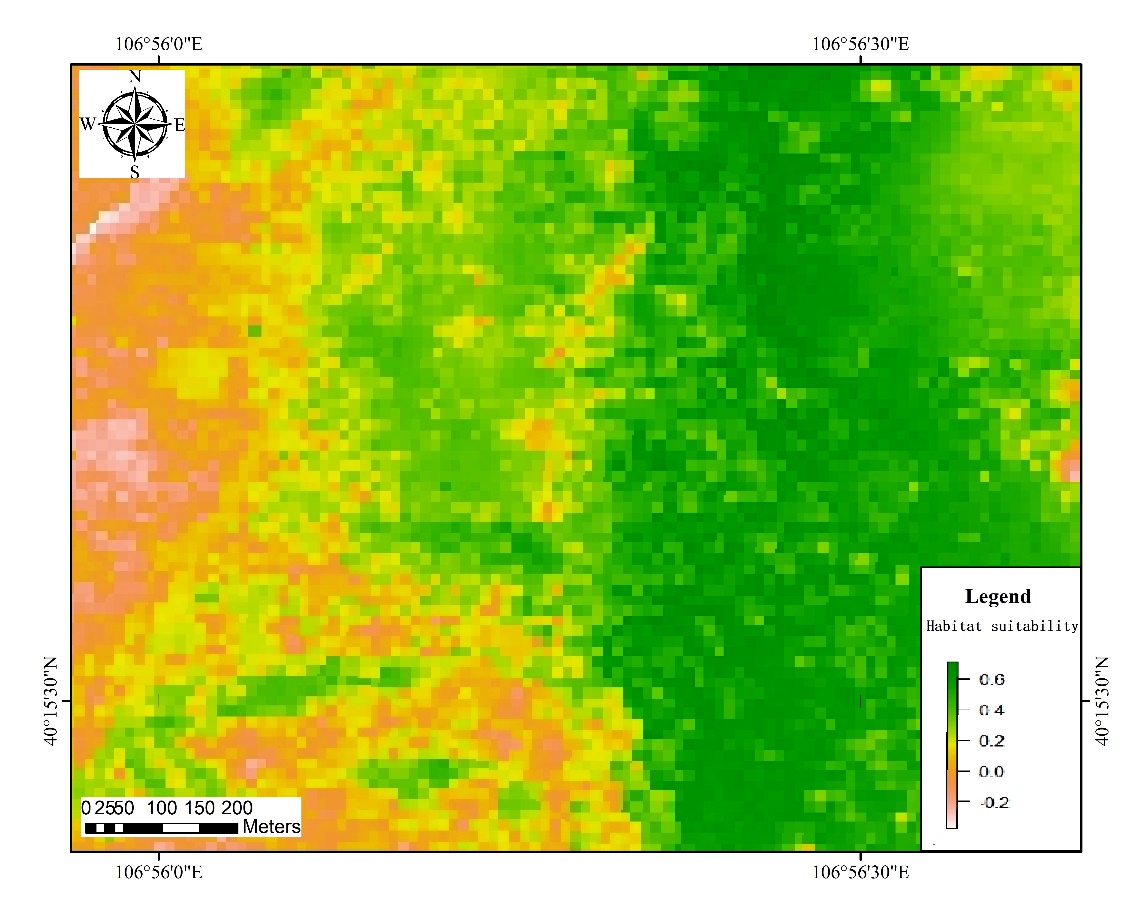


**Sup. Figure 4** habitat suitability map with GLM


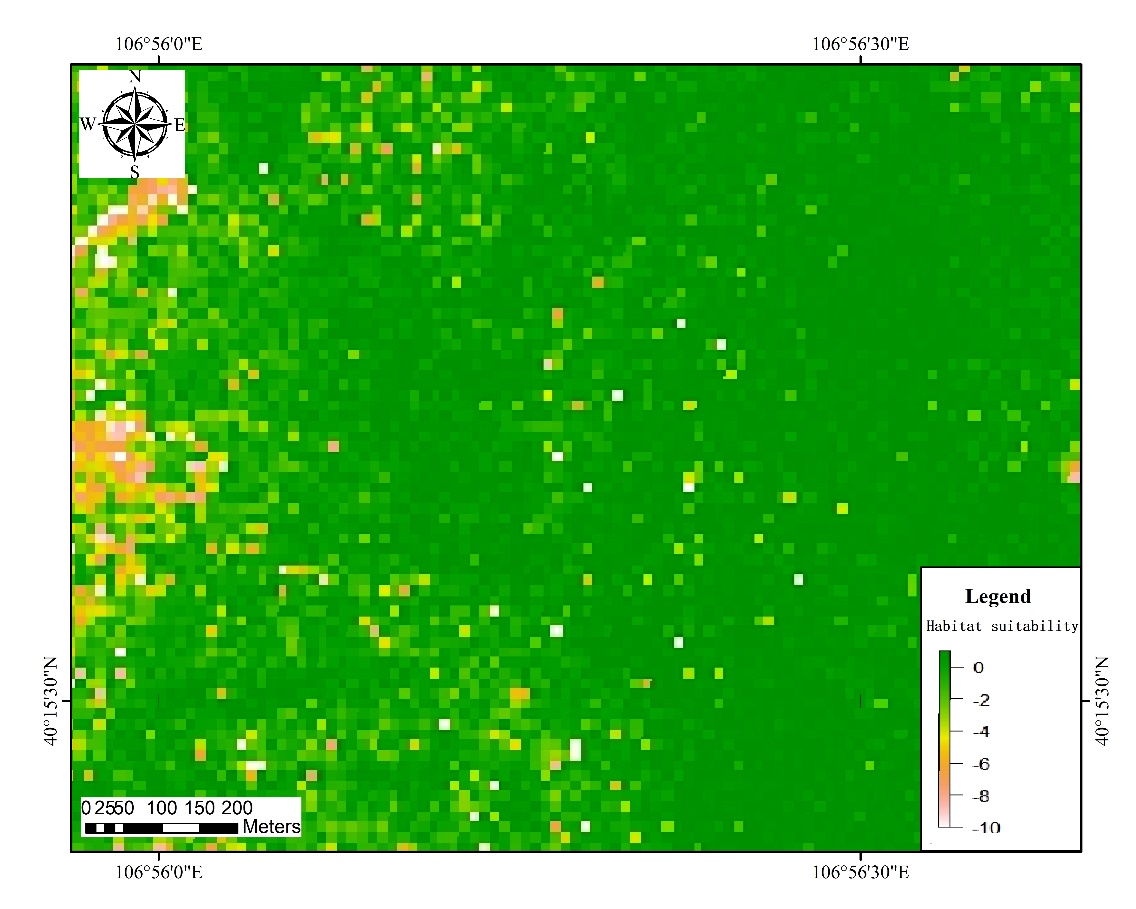


**Sup. Figure 5** Habitat suitability map produced with Mahalanobis


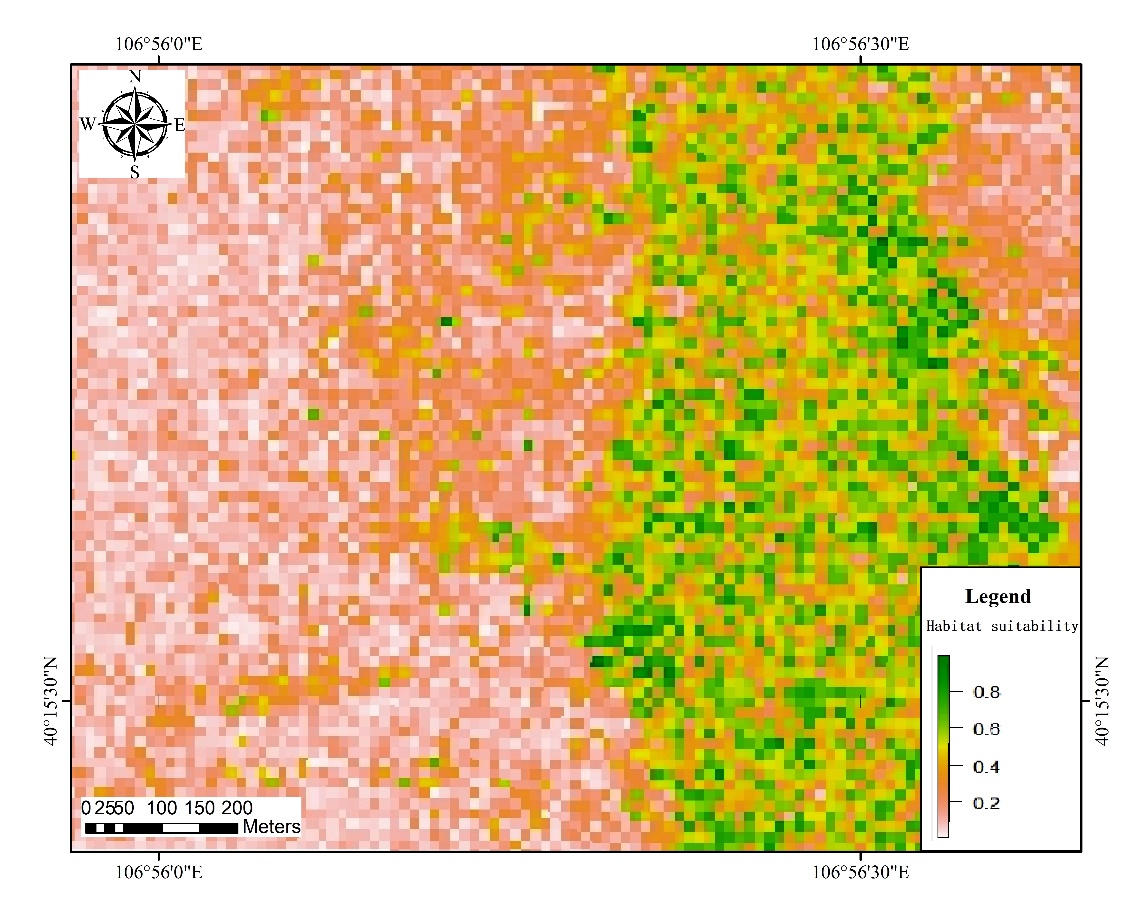


**Sup. Figure 6** Habitat suitability map produced with RF


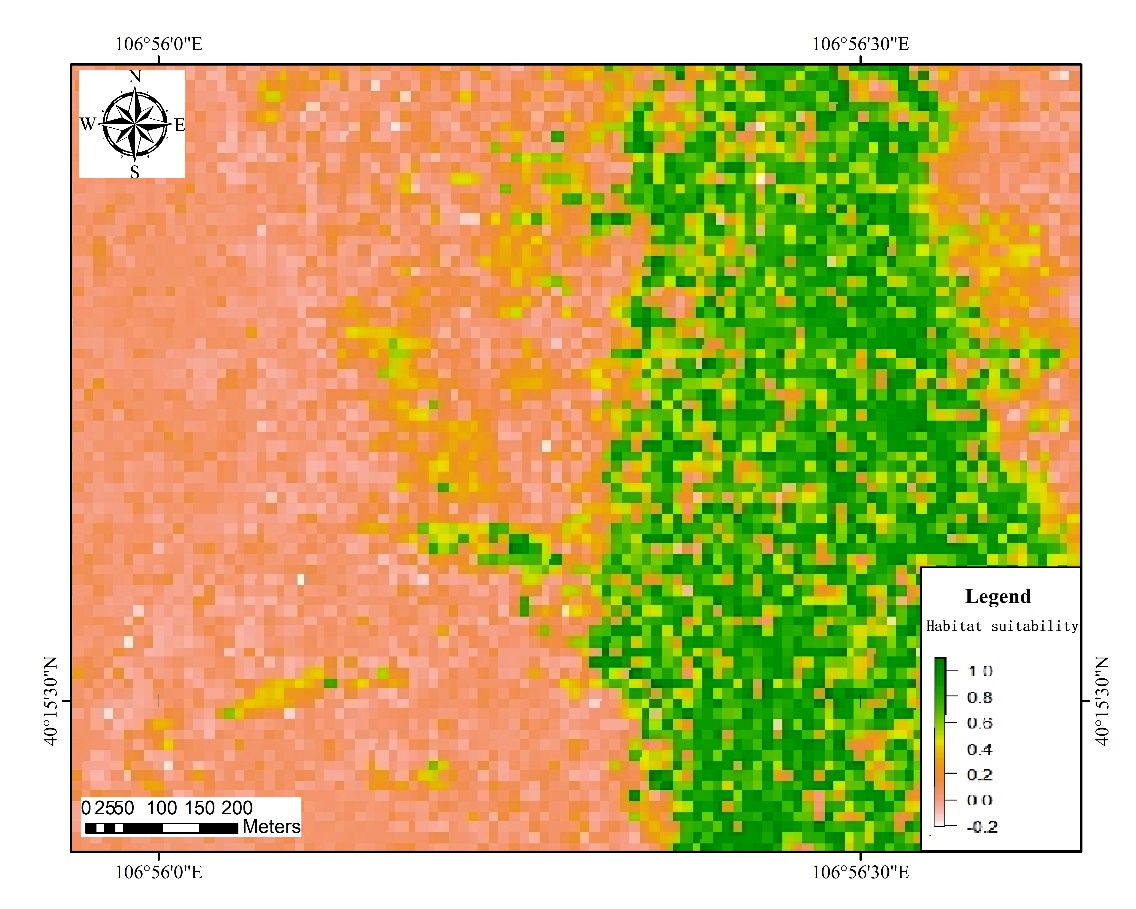


**Sup. Figure 7** Habitat suitability map produced with SVM


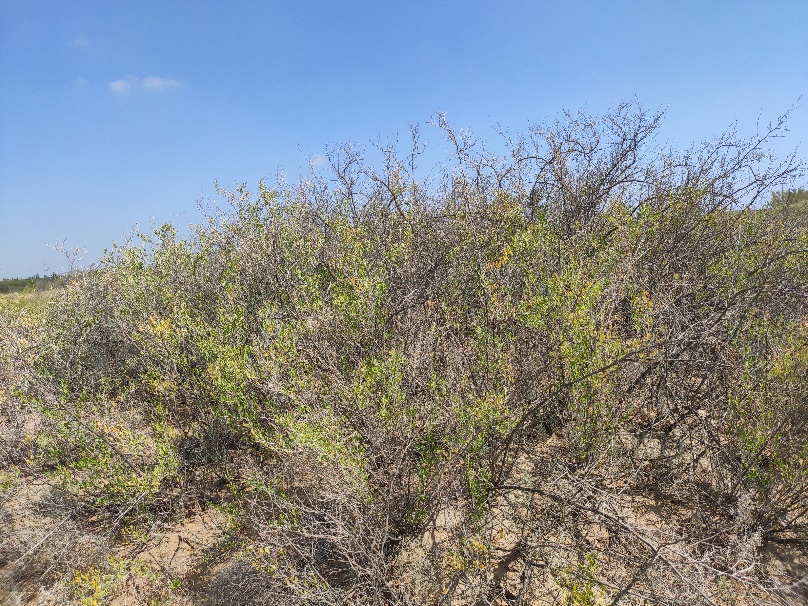


1 m

**Sup. Figure 8**  *Nitraria tangutorum* Bobr.

(N40°15′27.4248″,E106°56′5.2151″,1.5 m tall, contributed by Huoyan Zhou)
